# TRIM21—From Intracellular Immunity to Therapy

**DOI:** 10.3389/fimmu.2019.02049

**Published:** 2019-08-28

**Authors:** Stian Foss, Maria Bottermann, Alexandra Jonsson, Inger Sandlie, Leo C. James, Jan Terje Andersen

**Affiliations:** ^1^Department of Biosciences, Centre for Immune Regulation, University of Oslo, Oslo, Norway; ^2^Department of Immunology, Centre for Immune Regulation, Rikshospitalet, Oslo University Hospital and University of Oslo, Oslo, Norway; ^3^Department of Pharmacology, Institute of Clinical Medicine, University of Oslo and Oslo University Hospital, Oslo, Norway; ^4^Laboratory of Molecular Biology, Protein and Nucleic Acid Chemistry Division, Medical Research Council, Cambridge, United Kingdom

**Keywords:** TRIM21, antibody, gene therapy, virus, infection

## Abstract

Tripartite motif containing-21 (TRIM21) is a cytosolic ubiquitin ligase and antibody receptor that provides a last line of defense against invading viruses. It does so by acting as a sensor that intercepts antibody-coated viruses that have evaded extracellular neutralization and breached the cell membrane. Upon engagement of the Fc of antibodies bound to viruses, TRIM21 triggers a coordinated effector and signaling response that prevents viral replication while at the same time inducing an anti-viral cellular state. This dual effector function is tightly regulated by auto-ubiquitination and phosphorylation. Therapeutically, TRIM21 has been shown to be detrimental in adenovirus based gene therapy, while it may be favorably utilized to prevent tau aggregation in neurodegenerative disorders. In addition, TRIM21 may synergize with the complement system to block viral replication as well as transgene expression. TRIM21 can also be utilized as a research tool to deplete specific proteins in cells and zebrafish embryos. Here, we review our current biological understanding of TRIM21 in light of its versatile functions.

## Introduction

Antibodies are a crucial part of the immune response to invading viruses, and induction of neutralizing antibodies is a primary goal of vaccination (1). In addition, there is great interest in the isolation and engineering of broadly neutralizing antibodies against major human pathogens, such as human immunodeficiency virus and influenza virus for prophylactic and therapeutic uses (2–4). Antibody-mediated neutralization is generally considered to occur extracellularly due to exclusion of antibody from the cell interior by membrane compartmentalization. Extracellular neutralization has also been reported to be potentiated by engagement of Fc receptors (5, 6). However, the identification of tripartite motif containing-21 (TRIM21) as a high affinity cytosolic antibody receptor with anti-viral functions (7), has extended our understanding of the reach of antibody immunity to include the cytosol of cells.

Extracellular viral neutralization is thought to require a given level of antibody occupancy of specific epitopes to prevent entry into cells (8). However, viruses are known to display immuno-dominant epitopes that bias the polyclonal antibody response toward non-entry neutralizing epitopes (9–11). This implies that some viruses and bacteria have the capacity to penetrate the cell membrane and enter the cytosolic compartment even when they are opsonized with antibody. Such opportunistic pathogens are rapidly sensed by cytosolic TRIM21, which induces a synchronized effector and signaling response. Antibody-opsonized non-enveloped viruses are rapidly targeted for degradation via the proteasome and induce an innate immune response. Bacteria in complex with antibody trigger innate immune signaling (12) and possibly killing via autophagy (13). In both cases, TRIM21 functions as a link between the intrinsic cellular self-defense system and adaptive immunity by taking advantage of the diversity of the antibody repertoire to detect invaders (14). By doing so, TRIM21 distinguishes itself from other members of the TRIM protein family with anti-viral functions as these generally recognize the invading pathogen directly (15). TRIM21 recognition is also distinct from that of other innate sensors, such as pattern recognition receptors, which detects pathogen associated molecular patterns (PAMPs) (16). Instead, TRIM21 treats the displacement of antibody from the extracellular to the intracellular environment as a danger associated molecular pattern (DAMP) (12). Viral restriction by TRIM21 may also synergize with protective anti-viral mechanisms mediated by the complement system (17, 18).

**Figure 1:**
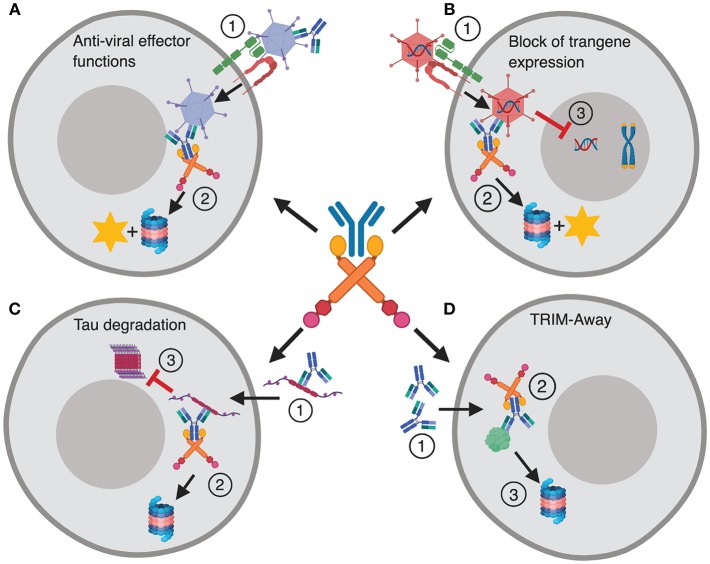
TRIM21 in disease and therapy. **(A)** The dual effector and signaling responses mediated by TRIM21 during Ad5 infection. (**1**) Ad5 coated with antibody enters the cytosol via CAR and αvβ3/5 integrin, (**2**) and is intercepted by TRIM21 that mediates proteasomal degradation and induces innate signaling. **(B)** TRIM21 mediated block to gene therapy. (**1**) An Ad5 based gene therapy vector enters the cytosol via CAR and αvβ3/5 integrin upon which (**2**) TRIM21 targets the vector for proteasomal degradation and induces innate signaling. (**3**) This hinders nuclear delivery and transgene expression. **(C)** (**1**) Tau protein bound by antibodies enters the cytosol via endocytosis and is targeted for destruction by TRIM21 (**2**). (**3**) This prevents formation of large intracellular tau aggregates. **(D)** TRIM-Away; (**1**) Antibodies are microinjected or electroporated into cells where they bind their antigen, (**2**) TRIM21 is recruited and directs the targeted protein to proteasomal degradation. The figure was made in BioRender™.

In addition to its role in intracellular defense, the implications of TRIM21 in therapy and as a research tool in cell biology are beginning to emerge. This includes identification of TRIM21 as a key player in preventing efficient adenovirus based gene delivery and vaccination (19), as well as proteasomal targeting of spreading tau protein (20). The latter promotes tau degradation instead of intracellular aggregation, which is associated with several neurodegenerative diseases. Finally, microinjection or transformation of antibody into cells can be used to direct intracellular proteins for TRIM21 mediated destruction. This technology is called TRIM-Away and is used to study protein function in cells (21, 22). These functions of TRIM21 are summarized in Figure 1.

In this review we discuss our current understanding of TRIM21 as a cytosolic Fc receptor that executes its effector functions alone or in synergy with the complement system. In addition, we highlight why TRIM21 should be considered in the context of disease and therapy.

## TRIM21 and Its Interaction With Antibody

**Figure 2:**
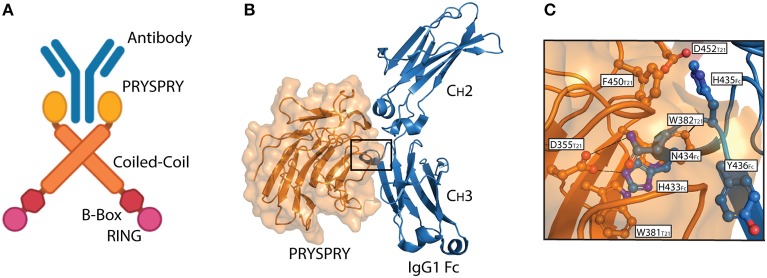
The TRIM21-IgG interaction. **(A)** Illustration showing binding of dimeric full-length TRIM21 to antibody (blue). The RING (pink), B-Box (red), Coiled-coil (orange), and PRYSPRY (yellow) domains of TRIM21 are shown. **(B)** Structural illustration of the interaction between the globular human PRYSPRY domain (orange) of TRIM21 and human IgG1 Fc (blue) (25). Insertion of Fc loop 429–436 into the PRYSPRY binding pocked is highlighted with a square. **(C)** Close-up view showing the insertion of IgG1 Fc loop 429–436 into the hydrophobic binding pocket on TRIM21 PRYSPRY. **A** was made in BioRender™ while **B,C** were made in PyMOL using PBD ID: 2IWG crystallographic data (25).

TRIM21 is a multi-domain protein consisting of an N-terminal RING domain with E3 ubiquitin ligase activity, a B-box domain, a coiled-coil dimerization domain and a C-terminal PRYSPRY domain (23). The domain architecture is conserved within the TRIM protein family and it is the C-terminal PRYSPRY domain that contains the antibody binding site and thus dictates function (24). The TRIM21 PRYSPRY domain is a globular fold comprising a β-sandwich of two anti-parallel β-sheets connected by flexible loops, which are sub-divided into PRY and SPRY elements (25, 26). In solution, TRIM21 exists as a homodimer and forms a stable 1:1 complex with antibody, in which the two PRYSPRY domains bind symmetrically to the Fc (7, 25). An illustration of dimeric TRIM21 in complex with antibody is shown in Figure 2A.

TRIM21 is also known as Ro52 and was first identified as a major autoantigen in autoimmune diseases such as Sjogren's syndrome and systemic lupus erythematosus (SLE) (27–29). Autoantibodies generated against TRIM21 in SLE are specific for the RING and B-box domains and not the Fc binding PRYSPRY domain (30).

Interaction between TRIM21 and part of an immunoglobulin heavy chain was first reported as a false positive in a yeast two-hybrid screen (31). Since then, direct binding between *bona fide* antibodies and TRIM21 has been demonstrated and its affinity and mechanism of binding dissected by site-directed mutagenesis and binding studies combined with solving the crystal structure of the human TRIM21 PRYSPRY domain in complex with a human IgG1 Fc fragment (25). The structure confirmed that two PRYSPRY domains bind to each side of the homodimeric Fc. The TRIM21 binding site is located at the C_H_2-C_H_3 interface of the Fc. This is distal from the binding site for the classical Fcγ receptors and complement factor C1q (32–36), but overlaps with that of the neonatal Fc receptor (FcRn) (37, 38), as well as viral and bacterial defense proteins (39–41) (Figure 2B). Notably, the TRIM21-IgG interaction is largely pH-independent and unaffected by removal of the bi-antennary *N*-glycan structure attached to N297 in the Fc C_H_2 domain (25, 31). It is, however, sensitive to high salt concentrations (25).

The core TRIM21-IgG1 interaction is formed between a protruding loop encompassing residues 429–436 in the Fc C_H_3 domain and a deep binding pocket formed on the surface of the PRYSPRY domain (25). The apex residues H433, N434, H435, and Y436 (HNHY-motif) of the Fc loop is inserted into the PRYSPRY binding pocket. The residues form a hydrogen bond network with the base of the pocket that is protected from solvent by a shield of hydrophobic side chains. Key interacting residues in the PRYSPRY domain include D355, W381, W383, D452, F450, and W299. A detailed view of the interaction is depicted in Figure 2C.

The binding affinity between human IgG1 and the recombinant human PRYSPRY domain has been measured to be in the range of 150–200 nM by both isothermal titration calorimetry and surface plasmon resonance (7, 42). However, since TRIM21 is homodimeric, its functional affinity upon binding symmetrically to the IgG1 Fc is as low as 0.6 nM as measured by fluorescence anisotropy (7). This represents an increase of >300-fold compared to monomeric binding, making TRIM21 the highest affinity Fc receptor known in humans.

The TRIM21-IgG interaction is highly conserved across species, which is illustrated by the fact that both human and mouse TRIM21 efficiently bind IgG from a range of mammals (26). There is also a strict correlation between site specific mutations made in mouse TRIM21 on binding to both mouse and human IgG subclasses, which is indicative of both thermodynamic and kinetic binding conservation across species. In line with this, TRIM21 distinguishes itself from other Fc receptors in that its PRYSPRY domain does not only bind all four IgG subclasses, but also IgM and IgA. The monomeric affinity of TRIM21 PRYSPRY to IgM and IgA are much weaker, 17 and 50 μM, respectively (7, 43). This is due to large differences in the amino acid composition of the Fc loop corresponding to the HNHY-motif in IgG, which is PNRV in IgM and PLAF in IgA. However, the loop is accommodated into the PRYSPRY binding pocket, and due to the dimeric nature of full-length TRIM21, functional affinity is likely to be in the sub-μM range (43). Both IgM and IgA have been shown to trigger TRIM21 effector functions (7, 43). Whether or not TRIM21 is able to interact with IgD or IgE has not yet been investigated, but nevertheless, it is the only known Fc receptor that is capable of interacting with three distinct antibody isotypes.

## TRIM21 Coordinates Viral Neutralization and Innate Signaling

How TRIM21 responds to antibody-coated viruses in the cytosol has been studied in detail using human adenovirus type 5 (Ad5) as a model pathogen. Upon infection of cells with Ad5-antibody complexes, TRIM21 mediates a sequential and coordinated effector and signaling response. This involves proteasomal degradation of the virus and induction of an anti-viral cellular state through activation of innate immune signaling pathways. The degradation of invading viruses by TRIM21 is termed antibody dependent intracellular neutralization (ADIN). This process occurs with rapid kinetics and can be observed with only a few antibodies per virus (44).

The dual effector response is fully dependent on the E3 ubiquitin ligase activity of TRIM21 mediated by its RING domain (45, 46). Upon engagement of antibody, TRIM21 is N-terminally monoubiquitinated by the E2 enzyme Ube2W. Then, the monoubiquitin acts as a primer for ubiquitin chain extension via a K63 linkage using the E2 enzyme complex Ube2N/Ube2V2. Depletion of cellular Ube2N leads to loss of both K63 and K48-linked ubiquitin from over-expressed TRIM21, suggesting that subsequent K48-linked ubiquitin may be incorporated, in the form of mixed or branched chains (45).

**Figure 3:**
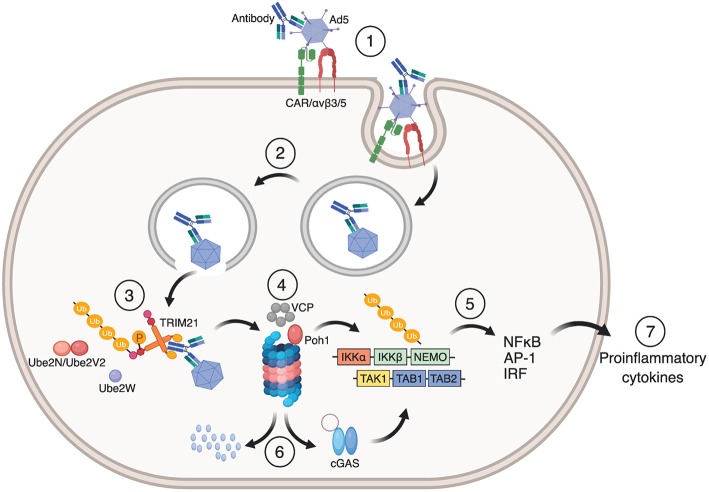
Mechanism of TRIM21 mediated anti-viral function. (**1**) Ad5 engages CAR and αvβ3/5 integrin at the cell surface. This triggers endocytosis of the Ad5:antibody complex and loss of fiber from the Ad5 capsid. (**2**) Fiber loss exposes protein VI that lyse the endosomal membrane which allows the virus:antibody complex to escape to the cytosol. (**3**) TRIM21 binds to the Fc part of the antibody in which auto-inhibition is released by B-box phosphorylation and undergoes auto-ubiquitination by the E2 enzymes Ube2W and Ube2N/Ube2V2. (**4**) This directs the Ad5:antibody complex to VCP and the proteasome for degradation. (**5**) Liberation of K63-linked ubiquitin chains by Poh1 activates IKKα-IKKβ-NEMO and TAK-TAB1-TAB2 which in turn induces NF-κB, AP-1 and IRFs resulting in the production of pro-inflammatory cytokines and an anti-viral state. (**6**) Exposed viral genomes trigger a second wave of immune signaling via the cytosolic DNA sensor cGAS. The figure was made using BioRender™.

Auto-ubiquitination of TRIM21 ultimately targets the TRIM21:antibody:antigen complex to the proteasome for degradation. Here, the proteasome associated deubiquitinase Poh1 liberates the ubiquitin chains *en bloc* to induce NF-κB, AP-1 and IRF 3, 5, and 7 signaling pathways via TBK1, TAB/TAK, and NEMO (12, 46). The result is production of pro-inflammatory cytokines. The ATPase p97/valosin-containing protein (VCP), an enzyme with segregase and unfoldase activity, is needed to degrade viral particles possibly because intact capsids have to be disassembled before the individual components are degraded in the proteasome (47). Virus degradation exposes viral genomes to the cytosolic DNA and RNA sensors cGAS and RIG-1, which initiates a second wave of immune signaling in response to Ad5 or human rhinovirus 14 (HRV-14) infection, respectively (48). The dual effector functions of TRIM21 are outlined in Figure 3.

TRIM21 contributes to systemic protection (49) *in vivo*. This has been demonstrated for mouse-adenovirus type 1 (MAV-1) infection, both in naïve mice and mice passively transfused with MAV-1 specific anti-serum. In these experiments, TRIM21 activity prevented MAV-1 induced hemorrhagic encephalitis as mice lacking TRIM21 had higher viral loads and increased mortality compared to wild-type (WT) animals, while mice heterozygous for TRIM21 displayed an intermediate phenotype. Interestingly, the fact that naive mice demonstrate TRIM21 dependent protection suggests that the early antibody response, likely in the form of IgM, works with TRIM21 *in vivo* to prevent infection. Also, use of the fully replicative MAV-1 demonstrates that TRIM21 is effective during an active spreading infection. Together, these experiments show that TRIM21 makes a substantial contribution to systemic protection and is an important part of the humoral immune response.

Recently, the role of TRIM21 in immune activation was mapped out in detail in a genome-wide differential gene expression analysis using RNA-seq (19). The analysis revealed that immune signaling is strictly dependent on TRIM21 and its binding to antibody. In TRIM21 knockout (KO) mice gene expression in naïve or Ad5 infected animals was barely affected. Similarly, infection in the presence of the Ad5 hexon specific mouse-human chimeric rh9C12 antibody resulted in more than 700 differentially expressed genes between WT and TRIM21 KO mice (19). Furthermore, Ad5 infection in the presence of an engineered version of rh9C12, the TRIM21 non-binding IgG1-H433A (42), resulted in a similar gene expression pattern as Ad5 infection alone. Thus, both genetic knockdown of TRIM21 and abrogation of the TRIM21-IgG interaction on the protein level, prevented immune activation. Importantly, the H433A mutation specifically ablates TRIM21 binding without impairing binding to other Fc receptors or affecting the serum half-life of the antibody within the timeframe of the experiments (19, 42, 50).

The specific contribution of TRIM21 to immune signaling was determined by comparing the transcriptional changes in immune genes induced upon infection in WT vs. TRIM21 KO mice using rh9C12-WT vs. rh9C12-H433A. TRIM21 specifically induced genes related to innate and intrinsic immunity as opposed to genes associated with a strong inflammatory response such as acute phase proteins. This indicates that TRIM21 is a potent positive immune regulator that focuses the anti-viral response toward intrinsic immunity.

## Regulation of TRIM21 Immune Signaling

Extracellular engagement of classical transmembrane-bound Fc receptors results in intracellular activation or inhibitory signal transduction (51, 52). Their regulation is dependent upon factors such as expression level, receptor cross-linking and the ratio between activating and inhibitory receptors, which together set the activation threshold. Likewise, innate immune responses inside cells must also be tightly controlled. Members of the TRIM protein family sense ligands entering the cytosol, but it is not well-understood how they trigger signaling. One regulatory mechanism that has been proposed is that TRIM proteins are activated by higher order assembly, based on early observations of their formation of so-called cytoplasmic bodies (53). The requirement for higher order assembly to drive ubiquitination and activity was first suggested for TRIM5α (54, 55). Assembly is thought to be required to allow RING domains to dimerize, as they are normally separated from each other in TRIM dimers by a long coiled-coil. What drives TRIM assembly is less clear. Engagement of retroviral capsids by TRIM5α can induce oligomerization and hexagonal assembly via B-Box interactions between multiple TRIM5α molecules (56). Mutations that disrupt dimerization inhibit its catalytic activity (57).

While RING dimerization is not required for E2 enzyme binding it is needed to form contacts with E2-charged ubiquitin. This has been demonstrated for TRIM25 as mutations at the RING dimerization interface disrupts its catalytic activity *in vitro* (58). Likewise, higher order assembly drives N-terminal K63-linked ubiquitination of TRIM5α, which is required for NF-κB activation and capsid disassembly (56). Furthermore, overexpression of TRIM5α in cells constitutively induces NF-κB activation in the absence of infection, likely due to spontaneous formation of cytoplasmic bodies (56). This in turn drives auto-ubiquitination, resulting in more rapid protein turnover by proteasomal targeting. However, such an activation strategy does not seem to be used by TRIM21 since its B-Box is auto-inhibitory (59). When TRIM21 is overexpressed in cells, no NF-κB activation or proteasomal degradation occurs in the absence of virus and antibody. Moreover, substitutions in TRIM21 corresponding to residues crucial for TRIM5α dimerization do not render TRIM21 catalytically inactive (59). These differences are observed despite the fact that the isolated RING domains of both TRIM5α and TRIM21 are highly catalytically active *in vitro*. So how is TRIM21 activation regulated?

Recent results have revealed that TRIM21 is auto-regulated via the B-Box domain, as deletion of the B-box increases the catalytic activity of its RING domain (59). This represents a novel function for the B-box domain. Structurally, this is explained by the fact that the B-box mimics and occupies the E2 ubiquitin ligase binding site on the TRIM21 RING domain, thereby preventing its auto-ubiquitination and activation of immune signaling. This was revealed by solving a crystal structure of a truncated form of TRIM21 containing only the RING and B-Box domains (59). These structural observations were confirmed by nuclear magnetic resonance (NMR) where E2 enzyme Ube2N bound to RING but not RING-B-box variants of TRIM21. Thus, for TRIM21 to respond to antibody bound virus in the cytosol a mechanism for reliving the inhibitory effect of the B-box must be in place.

The auto-inhibitory mechanism has been shown to be released by phosphorylation of a non-conserved serine residue at position 80 in the human TRIM21 RING domain by IKKβ or TBK1 kinases, driving B-box displacement from the RING (59). In line with this, stimulation of cells with poly(I:C) or infection with antibody coated Ad5 resulted in TRIM21 phosphorylation. S80 is part of a pLxSI like motif in the C-terminal end of the RING domain and located at the binding interface with the B-box. When the phospho-mimicking mutation S80E was introduced into the TRIM21 RING-B-box variant catalytic activity was restored. Cellular data support a role for phosphorylation, as infection with antibody-bound Ad5 or HRV-14 in cells expressing a S80A TRIM21 mutant did not activate NF-κB, while activation and cytokine production was restored in cells expressing the S80E variant. Direct evidence of B-box displacement and Ube2N binding to TRIM21 have been obtained by NMR using the S80E variant (59). Interestingly, a similar regulatory mechanism exists for unrelated innate immune adaptors such as mitochondrial anti-viral signaling protein (MAVS), stimulator of interferon genes (STING) and TIR-domain-containing adapter-inducing interferon-β (TRIF), where signaling is potentiated by serine phosphorylation of a similar pLxSI motif (60).

Strikingly, TRIM21 dependent Ad5 neutralization proceeds independently of the S80 phosphorylation state, suggesting that signaling has a higher activation threshold than neutralization (59). This is an important observation since both the neutralization and signaling arms of TRIM21 require ubiquitination, which suggests that neutralization can proceed at a basal level without innate signaling being triggered. This finding is consistent with earlier studies showing that while neutralization is efficient using engineered rh9C12 IgG1 variants with up to 100-fold reduced binding for TRIM21, NF-κB induction is ablated or severely diminished (42, 44). Furthermore, design of rh9C12 IgG1 variants with differences in *on*- and *off*- rate kinetics demonstrated that while slower *off*-rates generally correlate with efficient TRIM21 activity, its signaling function was abrogated faster by a reduction in *off*-rate compared to its neutralization function (61). This may allow for a low level of TRIM21 activity without inducing an anti-viral state, which might be important for clearing low levels of free antibody displaced into the cytosol.

An important question that remains to be answered is what role TRIM21 effector functions play in different cell types. Recently, Ad5 in complex with an Fc-engineered IgG1 rh9C12 variant with 100-fold improved binding to the TRIM21 PRYSPRY domain was reported to up-regulate the co-stimulatory molecules CD80, CD83, CD86, and HLA-DR as well as increase the production of pro-inflammatory cytokines by monocyte derived dendritic cells (62). This translated into enhanced cross-priming and activation of CD8^+^ T cells at high multiplicity of infection. In contrast, the ADIN activity was found to be unaffected. Although the exact intracellular mechanism responsible for T cell cross-priming remains to be determined, the data suggest that artificial antibodies bound to adenovirus increase the ability of dendritic cells to activate of CD8^+^ T cells, highlighting the usefulness of Fc-engineering.

Taken together, although the effector and signaling functions of TRIM21 are synchronized, they respond in a distinct and separable manner to antibody engagement, antigen binding and auto-ubiquitination. The more stringent requirement for activation of immune signaling compared to neutralization serves to preserve an important hallmark of immunity, which is to elicit a balanced and proportionate response. If this regulation fails, the consequence of constitutive TRIM21 activation would likely be to trigger chronic inflammation. This has been demonstrated in lysosome-maturation impaired macrophages from lupus-prone mice, in which leakage of IgG containing immune complexes from the endosomal pathway into the cytosol triggers a TRIM21 dependent inflammatory response in the absence of infection (63).

## Susceptible Targets for TRIM21

The anti-viral function of TRIM21 has been studied using several viruses including Ad5, MAV-1 and human rhinovirus 14 (HRV-14) (7, 48, 49). In addition, it has been shown that porcine TRIM21 is able to restrict Foot and mouth disease virus (64). As the requirement for TRIM21 activation is entry of an antibody-coated particle into the cytosol, it is believed that enveloped viruses are not targeted by the receptor since they leave their membrane along with bound antibodies at the cell surface upon fusion with the plasma membrane. This has been demonstrated for human respiratory syncytial virus (RSV) bound by the therapeutic monoclonal IgG1 antibody palivizumab (Synagis®) (12). Other non-enveloped viruses, such as HRV-2, form a pore in the endosomal membrane through which their genomes are injected so that its capsid and any attached antibody cannot be sensed by TRIM21 (48). The paratope of the antibodies as well as the epitope on the virus may also determine whether or not it can be targeted by TRIM21. In case of Ad5, the most studied virus in this context, its capsid fiber protein is shed during endocytosis via αvβ3/5 integrin and the coxsackie and adenovirus receptor (CAR) and as such does not follow the rest of the viral particle into the cytosol (65). Therefore, a monoclonal Ad5 fiber specific antibody poorly recruits TRIM21 (19). However, most of the antibodies in polyclonal sera are not entry blocking and target epitopes on the immunodominant major capsid protein hexon (10, 19, 66–69). Interestingly, TRIM21 sensing is not limited to viral infection, as signaling may also be triggered in response to antibody-coated bacteria, such as *Salmonella enterica* (12). In addition, non-pathogenic complexes such as host-derived proteins bound by antibody or antibody-coated beads are also targeted by TRIM21 and activate its dual effector functions (20, 47).

## Implications for Therapy

Adenoviruses are major targets for TRIM21. In gene therapy, the objective is to deliver gene variants or genes encoding vaccine epitopes for expression in host target cells (70). Adenoviruses, and in particular Ad5, is frequently used as a vehicle for these transgenes (71). However, since adenoviruses are common pathogens in humans, pre-existing antibody immunity against the virus severely limits its use as it often prevents expression of the transgene (72). Notably, normal human serum may contain as much as 100 μg/ml of Ad5 specific IgG (18). Thus, Ad5 based gene delivery vehicles will be coated with antibodies after intravenous injection (i.v.), which may explain why CD8^+^ T-cell responses against Ad5 delivered vaccine antigens are poorly induced (72–75). However, finding a biological explanation for why the presence of antibodies blocks gene expression has been a puzzle because they do not prevent delivery of the transgene into the cytoplasmic space (73).

Recently, a substantial proportion of this antibody inhibition was demonstrated to be mediated by TRIM21 (19, 76). When naive mice were infected with Ad5 carrying a luciferase encoding transgene in the presence of the rh9C12 IgG1 antibody, expression of the transgene in the liver was reduced by 1000-fold compared to when the virus was administered alone. Transgene expression was not affected by antibody in TRIM21 KO mice, or in WT mice using the TRIM21 non-binding mutant rh9C12-H433A. This TRIM21 dependent block to transgene expression was also observed using polyclonal mouse serum raised against Ad5. Notably, potential re-routing of virus via classical FcγR mediated uptake was not of significant importance, as infection in the presence of a rh9C12 IgG1 variant that does not bind FcγRs (L234A/L235A) did not affect the block to transgene expression (19).

Further, impaired CD8^+^ T-cell responses against the SIINFEKL epitope (SL8) of ovalbumin (OVA) were demonstrated to be dependent on TRIM21 when mice were vaccinated with Ad5 carrying the OVA transgene in the presence of antibody (19). This resulted in reduced antiviral immunity, which was established using an engineered influenza virus strain (H1N1) carrying the SL8 peptide in its neuramidase stalk as well as reduced anti-tumor immunity as shown by challenge of Ad-OVA immunized animals with the OVA secreting fibrosarcoma cell line MCA101.

In addition to blocking transgene expression, induction of unwanted innate immune signaling may occur upon administration of adenovirus based gene delivery vectors (73, 77, 78). This may be related to the activity of TRIM21. Pre-clinically, this is supported by studies in mice infected with Ad5 in the presence of rh9C12 IgG1, where amplified transcription levels of pro-inflammatory NF-κB inducible cytokines, type-1 IFN and IFN stimulated genes (ISGs) were measured in the liver. The response was comparable to that of CpG, indicating biologically relevant induction levels. Clinical gene therapy trials indicate that pre-existing immunity to Ad5 vectors can be circumvented by intramuscular or intranasal delivery (79, 80). When Ad5 vector was given intranasally TRIM21 did not block OVA transgene delivery as potently as seen for i.v. delivery. However, this was accompanied by a 10-fold weaker OVA-specific CD8^+^ T-cell response (19). Insights into how blockade of transgene expression delivered by adenovirus vectors is mediated opens up for development of strategies to circumvent targeting of gene therapy vectors to TRIM21. Given the reduced T-cell response associated with alternative delivery routes, a vector shielding strategy to avoid pre-existing immunity may be the most advantageous approach.

While TRIM21 negatively impacts gene therapy, the activity of the receptor may be favorably utilized for other therapeutic applications. One interesting area is neurodegenerative disorders, such as Alzheimer's disease, which are characterized by aggregation of the intracellular microtubule-associated tau protein (20). Intracellular tau aggregates are generated from extracellular tau and can spread between cells (81, 82). Administration of tau specific antibody is known to reduce tau induced pathology in mice (83–86), but the underlying mechanism has not been elucidated yet. Interestingly, TRIM21 has been shown to inhibit intracellular tau aggregation in a newly established *in vitro* tau seeding assay that recapitulates tau aggregation in diseased brains (20). Interception of extracellular tau by anti-tau antibody in the extracellular environment during cell-to-cell transfer resulted in TRIM21 dependent proteasomal degradation. As tau aggregates fast after cytoplasmic entry, the rapid recruitment of TRIM21 to incoming antibody:tau complexes are likely crucial for prevention. This discovery may foster development of new therapeutic approaches where delivery strategies for antibodies through the blood-brain barrier could be combined with targeting of extracellular tau to TRIM21 (20, 87, 88).

## TRIM-Away

The ability of TRIM21 to target cytosolic proteins tagged by antibody for degradation can be utilized in research. This has recently been demonstrated by a technology that has been coined TRIM-Away (21). The method relies on introducing antibody, specific for an intracellular protein of interest, into single cells by microinjection or into cell cultures by electroporation (21, 22). The principle has been exemplified by microinjection of anti-GFP antibody into the human cell line NIH 3T3 over-expressing free GFP, which led to degradation of GFP within a few minutes (21). Likewise, when GFP was fused to a lipid or endogenous proteins and localized to distinct places within primary mouse oocytes, such as the plasma membrane, endosomal membranes and the nucleus, GFP was efficiently targeted by TRIM21. The depletion of nuclear proteins was dependent upon antibody access to the nucleus, and in non-dividing cells did not take place. One approach to solve this problem is to use alternative, smaller, antibody formats, for instance an Fc fragment fused to an anti-GFP nanobody that facilitated efficient nuclear transport and target degradation (21). The robustness of the method has been further studied in mouse oocytes, where targeting of the nuclear Eg5 protein resulted in rapid degradation that prevented bipolar mitotic spindle formation (21). Yet another example is targeted depletion of the long-lived Rec8 protein in mouse eggs that led to separation of sister chromatids. Importantly, this latter example addresses a major challenge, namely that long-lived proteins such as Rec8 cannot be effectively depleted using standardized DNA or RNA based methods due to their very slow turnover rate in cells (89, 90). Finally, TRIM-Away allows the depletion of specific proteins in primary human cells. This was demonstrated by depleting IKK and NLRP3 in primary human macrophages. Depletion of NLRP3 was also shown to functionally alter the macrophages, making them resistant to pyroptosis and reducing their release of potent inflammatory cytokine IL-1β (21, 91). TRIM-Away has further been used to demonstrate involvement of SNAP23 in meiotic arrest and regulation of exocytosis in developing mouse eggs (92). The usefulness of TRIM-Away to manipulate primary cells is highlighted by the fact that other approaches, such as transfection or transduction of RNA or DNA, are inefficient and stimulate innate signaling (93, 94).

The duration of knockdown by TRIM-Away correlates with the amount of antibody and TRIM21 present (21). TRIM21 is saturable when cells are exposed to high viral loads in complex with antibody (44), which is explained by its expression level, but also in part by the fact that TRIM21 is degraded together with the antibody bound target. Despite TRIM21 being IFN-inducible, which is likely crucial to sustain its activity during an ongoing infection, the expression level of TRIM21 is in many cases a limiting factor for the TRIM-Away strategy. This is especially true when abundant cellular proteins are targeted but can be overcome by over-expression or co-administration of recombinant TRIM21 during microinjection or electroporation. Importantly, over-expression of TRIM21 does not alter the cellular transcriptome, cell phenotype or endogenous levels of other proposed TRIM21 ligands (21). When the centrosome protein percentrin was targeted in NIH 3T3 cells over-expressing TRIM21, there was efficient depletion, and percentrin failed to localize with its nuclear interaction partner Cdk5rap2. In addition, the method has been used to either activate or inhibit signal transduction pathways by targeting its different components (21). Recently, the TRIM-Away method was also reported to be an attractive tool to degrade or delay expression of proteins in zebra-fish embryos, thus enabling investigation of how specific proteins affect embryonic developmental processes (95).

A major advantage of TRIM-Away is that it is rapid and allows for monitoring of phenotypic changes in cells while at the same time limiting the appearance of compensatory mechanisms (21). On the other hand, it relies on the use of antibodies that are highly specific and do not cross-react with other intracellular proteins. A potential issue that should be taken into consideration is the possibility that targeting of one cellular protein may result in the concomitant depletion of strong interaction partners. However, such knock-on effects are also likely to result from reduced expression of the target via siRNA or shRNA or gene KO via CRISPR Cas9.

## TRIM21 Synergy With Complement

**Figure 4:**
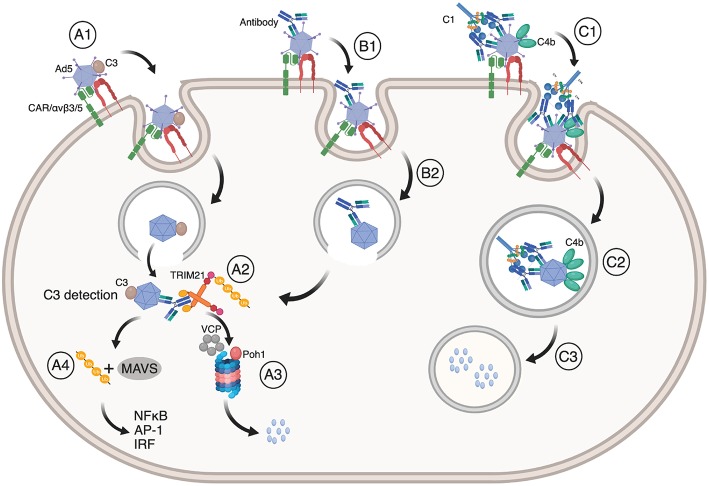
TRIM21 synergizes with the complement system. (**A1**) Ad5 with deposited C3 enter cells via CAR and αvβ3/5 mediated endocytosis, upon which (**A2**) Ad5:C3 complexes escape into the cytosol where C3 is detected. This may occur synergistically with TRIM21 engagement (**B1, B2, A2**). In both cases, Ad5 is directed to proteasomal degradation (**A3**) followed by ubiquitin or MAVS mediated induction of NF-κB, AP-1, and IRFs (**A4**). (**C1**) Antibody coated Ad5 bound by C1 may result in C4b deposition on Ad5 prior to endocytosis via CAR and αvβ3/5 integrin. (**C2**) C4b prevents exposure of the Ad5 membrane lytic protein VI and blocks endosomal escape. Instead, Ad5 is routed into lysosomes where it is degraded (**C3**). The figure was made using BioRender™.

The immune system orchestrates a range of effector mechanisms to protect against infection. In humans, the complement system consists of more than 20 proteins that label pathogens for destruction via the classical, mannose lectin or alternative pathways (96, 97). After labeling, viruses may still enter endosomal compartments as well as the cytosol. As such, complement may synergize with TRIM21 in different ways to prevent cellular infection of non-enveloped viruses. These mechanisms are illustrated in Figure 4. This was first demonstrated by C3 deposition on the virus surface via the alternative complement pathway in a factor B and D dependent manner (17). C3 deposition triggers a dual effector response similar to that mediated by TRIM21 in the cytosol. C3 coated Ad5 is targeted to the proteasome in a VCP dependent manner where it is degraded and also induces innate signaling via NF-κB, AP-1 and IRF in non-hematopoietic cells. Further, C3 mediated immune activation may synergize with TRIM21 and antibody dependent signaling (7, 12). The efficacy of C3-dependent signaling varies among different viruses such as Ad5, HRV-14 and poliovirus P2, in which the strength of the response correlated with the degree of endosomal lysis and escape of intact C3 bound capsids into the cytosol (17). Again, enveloped viruses, such as RSV, do not efficiently trigger the cytosolic effector response.

The intracellular receptor for C3 has not yet been identified, but is likely to involve an IFN inducible gene, as C3 dependent neutralization of Ad5 is potentiated by IFN stimulation (17). The C3-dependent signaling response is dependent on MAVS and proceeds through the TNF receptor-associated (TRAF) pathway. MAVS is not required for C3 dependent neutralization which suggests that initial C3 sensing occurs via an upstream component.

While no antagonistic mechanism has been identified for TRIM21, certain viruses, such as HRV, produce cytosolic 3C proteases that cleave C3 as a protective mechanism. This has been demonstrated for HRV-14, where the 3C protease disables both signaling and degradation in the cytosol of non-hematopoietic cells, a phenotype that could be reversed by the protease inhibitor rupintrivir (17). The fact that TRIM21 uses antibody to sense pathogens, as opposed to direct deposition of C3, suggests that it is more difficult for the virus to directly antagonize it. On the other hand, some non-enveloped viruses inject their viral genome into the cytosol via pores formed in endosomal membranes, which separates antibody from TRIM21 and as such can be considered a viral protective strategy (98).

In addition to direct deposition of C3 on viral capsids via the alternative pathway, antibody opsonized pathogens may recruit the C1 complex (C1qC1r_2_C1rs_2_) to initiate the classical complement pathway. Recruitment of C1 results in cleavage of C4 followed by association of C4b with C2a to form the C3 convertase that again cleaves C3 leading to C3b opsonization and downstream formation of the membrane attack complex (MAC) (96, 97). Recently, neutralization of Ad5 was shown to occur not only via the antibody-TRIM21 axis, but also via the classical complement pathway (18). This neutralization mechanism depends on binding of C1 to antibody-opsonized Ad5 and subsequent C4b deposition on the virus, which occurs independently of other complement components.

Ad5 infection is a stepwise process that progresses from binding to the host cell receptors to endocytosis and lysis of the endosomal membrane (99–105). Ad5 engages two host cell receptors, CAR and αvβ3/5 integrin, and these binding events provide mechanical cues for the virus to commence its stepwise uncoating process. This results in loss of the fiber protein and exposure of the membrane lytic protein VI, which lyses the endosomal membrane. Upon entry into the cytosol, hexon recruits dynein and the capsid moves toward the nucleus (105–109). During this process, the capsid continues to progressively disassemble into a more meta-stable state that ultimately serves to deliver the viral genome into the nucleus. However, when C4b is deposited on an antibody coated virus, it interferes with viral disassembly in the endosome since the membrane lytic protein VI is not exposed (18). As a consequence, the virus is routed down the endo-lysosomal pathway and does not reach the cytosol. In experiments where Ad5 was opsonized with a mutant rh9C12 IgG1 variant (P329A) that fails to interact with C1, the virus readily escaped into the cytosol and is instead targeted by TRIM21. Thus, TRIM21, C1, and C4, and likely C3 dependent neutralization pathways may operate synergistically during Ad5 infection (17, 18). This raises the question as to which of these neutralization mechanisms dominates under different conditions.

For C4b to be deposited on the surface of Ad5 to an extent that results in C1C4 dependent neutralization, roughly 25 rh9C12 IgG1 antibodies must bind the virus (18). The EC_50_ of C4 deposition has been determined to be 45 μg/ml, which is 10-fold lower than normal serum concentrations. As the Ad5 specific IgG titer is around 100 μg/ml in normal human serum, this strongly indicates that C4 mediated neutralization of Ad5 is likely to be a significantly contributing factor *in vivo*. This was confirmed in mice infected with Ad5 expressing a luciferase transgene where expression of the transgene was inhibited in the presence of rh9C12 IgG1, but partially restored in C4 KO mice (18). The remaining block to transgene expression was TRIM21 dependent, consistent with what is observed *in vitro*. When TRIM21 KO mice were given Ad5 in the presence of rh9C12-P329A, transgene expression was fully restored to control levels. This effect was confirmed in WT mice vaccinated with Ad5-OVA together with the double negative rh9C12 mutant IgG1-P329A/H433A that does not bind TRIM21 nor C1q, as CD8^+^ T-cell induction was completely restored 10 days post administration. Thus, the complement system and TRIM21 may work in concert to prevent infection but also to block Ad5 based gene delivery.

## Antibody Engineering Boosts Gene Therapy

Insights into how pre-existing antibodies limit the efficacy of gene therapy, may pave the way for strategies to circumvent the problem. One conceivable strategy may be to pre-coat Ad5 vectors with an antibody engineered to ignore both C1q and TRIM21, such as P329A/H433A, or simply use a Fab fragment. Using an *in vitro* competition assay, where Ad5 infection was measured in the presence of a constant amount of rh9C12 IgG1 and increasingly higher concentrations of rh9C12-P329A/H433A or Fab, both strategies were shown to interfere with complement mediated neutralization at low concentrations (18). In contrast, much higher concentrations of the competitors were required to prevent TRIM21 mediated neutralization. This reflects that TRIM21 neutralization only requires a few antibodies bound to the virus to be effective (44), while efficient C1C4 neutralization requires higher antibody coating levels (18). When WT mice where infected with Ad5 carrying the luciferase transgene in the presence of a 1000-fold excess of a rh9C12 derived Fab fragment, a 10-fold improvement in transgene expression was observed. Importantly, the Fab pre-coating strategy also diminished the polyclonal antibody response toward the vector when administered intramuscularly to naïve mice, suggesting that this strategy could also prevent a protective antibody response during repeated vector dosing.

As hexon is the primary immunogen to which antibodies is generated, rh9C12 that binds to the apex of the hexon trimer spike (61), may be particularly well suited for such pre-coating. Indeed, shielding the Ad5 surface with a trivalent 9C12 scFv with high avidity has been used in combination with targeted designed ankyrin repeat proteins (DARPin's) binding to the fiber knob of an Ad5 vector (110). The DARPin units block CAR mediated uptake and re-targeted the vector to HER2 or EGFR positive tumor cells in mice (110, 111). This combination strategy improved the tumor to liver localization ratio of the Ad5 vector and circumvented intracellular neutralization by TRIM21, thus significantly boosting transgene expression.

## Concluding Remarks

The role of TRIM21 as a high affinity cytosolic Fc receptor has grown in significance in both disease and therapy. The use of genetic KO of TRIM21, both *in vitro* and in mice, together with specific KO of the interaction on the protein level using engineered antibody variants have solidified the role of TRIM21 as a positive immune regulator whose activity is strictly dependent on detection of cytosolic antibody-virus complexes (19). It should be noted however, that TRIM21 has also been implicated in both positive and negative regulation of innate signaling independent of antibody binding (112–117). Furthermore, the identification of TRIM21 as a major player in antibody mediated block to gene therapy, together with the effect of complement factors C1 and C4, have inspired strategies to shield Ad5 gene delivery vectors from pre-existing immunity (19, 110). The finding that such shielding strategies limit immune responses to the vector should be of particular interest in cases where repeated administrations are needed.

Moreover, the remarkable ability of TRIM21 to engage three different antibody isotypes (7, 43) is in sharp contrast to other Fc receptors that are highly selective in regard to both isotype and subclass binding properties. Early primary immune responses to infections are dominated by IgM while IgG emerges later and in secondary responses upon re-infection. The IgG response is initiated by IgG3 followed by IgG1, both of which are considered anti-viral subclasses (118–121). However, how each of the four human IgG subclasses activate TRIM21 in the cytosol when bound to viruses has yet to be addressed in detail. Furthermore, since TRIM21 may synergize with the complement system, it will be important to fully understand how anti-viral immunity is orchestrated by different antibody isotypes and subclasses in the absence and presence of C1/C4.

Even though circumvention or utilization TRIM21 activity for therapeutic purposes are at the concept stage, the impact of alleviating the block to adenovirus based gene therapy or using antibody and TRIM21 to prevent intracellular tau aggregation are potentially huge. If a method to deliver antibody into the cytosol of specific cells *in vivo* with sufficient efficacy was to be developed, one could even imagine that the TRIM-Away technology could be exploited therapeutically to specifically degrade intracellular disease associated proteins (21).

Furthermore, TRIM21 function has been extensively studied using Ad5 as a model pathogen. While adenovirus infection in healthy individuals is generally benign, it may cause severe complications and even death in immunocompromised or immunosuppressed patients (122). For example, aggressive adenovirus infections in the human eye, known as *epidermic keratoconjunctivitis*, may lead to loss of vision to which there are no available treatment options (123). Whether adenovirus infections in the eye may benefit from TRIM21 and antibody therapy remains to be addressed, however, injection of antibodies into the vitreous is frequently used in the clinic to treat eye diseases such as age-related macular edema (124, 125). While activation of a strong inflammatory response may not be beneficial in a treatment setting, using engineered antibodies with reduced affinity for TRIM21 or altered antigen binding kinetics could be attractive to reduce inflammation without compromising virus neutralization (42, 61).

The ability of TRIM21 to take advantage of the antibody repertoire and orchestrate a potent intracellular immune response to invading pathogens provides non-hematopoietic cells the means to actively protect themselves even after a virus have entered the cytosol. However, as TRIM21 is also expressed by professional immune cells, its role in these cell types and how it affects antigen processing and presentation in the context of other antibody binding receptors will be important to address.

## Author Contributions

All authors listed have made a substantial, direct and intellectual contribution to the work, and approved it for publication.

### Conflict of Interest Statement

The authors declare that the research was conducted in the absence of any commercial or financial relationships that could be construed as a potential conflict of interest.
